# Exploring the inside details of virions by electron microscopy

**DOI:** 10.1007/s41048-016-0022-7

**Published:** 2016-04-22

**Authors:** Zheng Liu, Jingqiang Zhang

**Affiliations:** 1 Department of Biochemistry and Molecular Biophysics, Columbia University, New York, NY 10032 USA; 2 College of Life Science, SunYat-sen University, Guangzhou, 510275 China

Spherical virions can be structurally divided into two compositions: the outer protein shell, and its content including the viral genome and enzymes. Three-dimensional virus structures are the key to understanding the virus life cycles and their mechanisms for infecting hosts, which can contribute to anti-viral drug design. These structures can be solved either by X-ray crystallization (Harrison et al. 1978) or by cryo-electron microscopy (cryo-EM) (Dubochet et al. 1988), or by combining both methods (Guu et al. 2009). In retrospect, the X-ray method had begun to solve viral structures at high resolution with atomic information since the late 1970s (Harrison et al. 1978; Abad-Zapatero et al. 1980), while cryo-EM, considered as a blobology method, only provided low- or medium-resolution information from the 1980s (Dubochet et al. 1988). In recent years, cryo-EM has emerged to rival with X-ray on the high-resolution structure determination of spherical viruses (Grant and Grigorieff 2015; Liu et al. 2016). In spite of a wealth of virus structures determined by these methods, the solved parts were limited to the viral capsid, while the remaining moieties, like the viral genomes and virus-self-functional enzymes within, were rarely visualized. Strikingly, in a recent paper published in *Science* (Liu and Cheng 2015), Drs. Liu and Cheng managed to discern the full genome and polymerase of cytoplasmic polyhedrosis virus (CPV), using their new approach adapted from traditional single-particle analysis of icosahedral viruses.

**Fig. 1:**
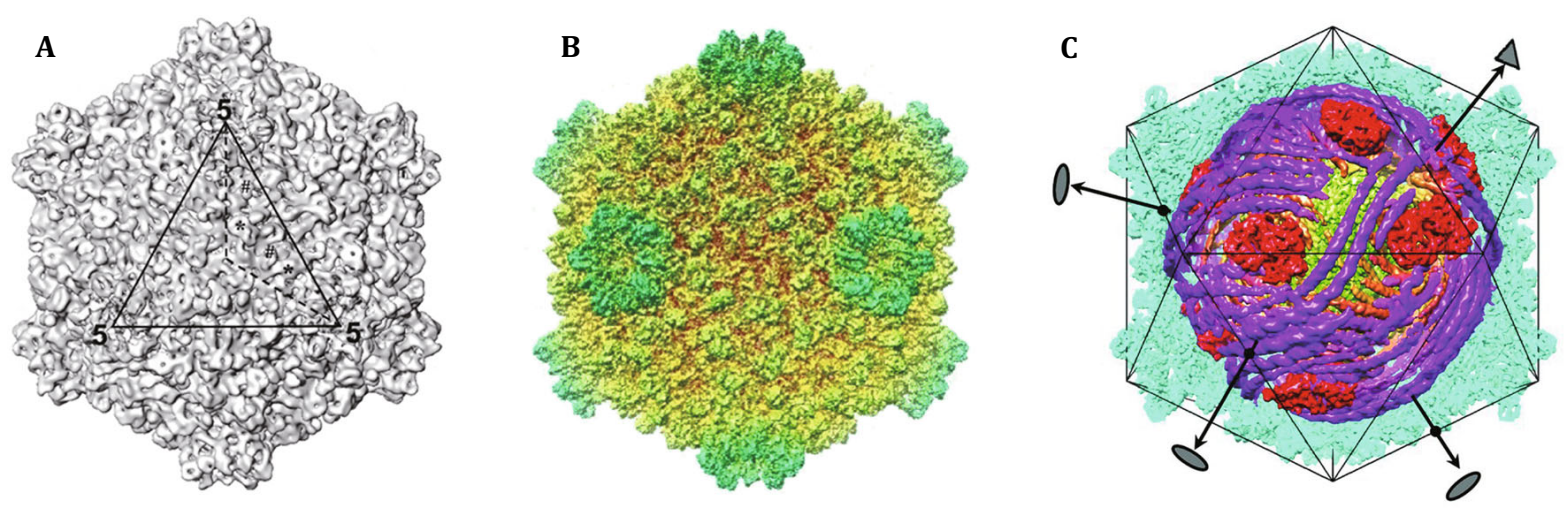
Cryo-EM of CPV. **A** A low-resolution structure of CPV (Zhang et al. 1999). **B** The 3.88-Å CPV structure, the first high-resolution macromolecule structure determined by single-particle cryo-EM (Yu et al. 2008). **C** The CPV structure reconstructed by symmetry-mismatch reconstruction (Liu and Cheng 2015)

The CPV belongs to the *Reoviridae* family, whose members cause diverse diseases in their hosts ranging from plants, insects, to humans. CPVs cause diseases only in arthropods and therefore are considered as potential biological insecticides to combat their hosts injuries to agriculture. The *Bombyxmori* CPV (BmCPV), the exact species used in the presented study, can cause serious flaccidness in silkworm, posing a big burden in sericulture development in East Asia. The genome of BmCPV is composed of 10 segments of double-stranded (ds) RNA. The naturally mature virions encapsulate their own RNA-dependent RNA polymerases (RdRps) into the capsid and transcribe the minus-strand RNA to mRNA inside the intact capsid, which usually occurs in host cell’s cytoplasm. In comparisons with other members of *Reoviridae* having a *T* = 13 layer (here *T* is the triangulation number), CPV virion only has a single layer capsid, adopting *T* = 1 lattice, with ~70 nm in diameter (Fig. 1A). Purification of virus from the silkworm midgut usually yields two kinds of particles—empty capsids and complete virions. Treated with GTP properly, the virions performing transcription (transcribing CPV, TCPV) can also be obtained from their non-transcribing state (non-transcribing CPV, NCPV).

To reveal the structure of CPV, both X-ray and single-particle cryo-EM were applied. Probably due to the intrinsic flexibility of some viral components, CPV had eluded the efforts of the X-ray method, while cryo-EM advanced the structural studies while steadily improving resolutions (Hill et al. 1999; Zhang et al. 1999; Xia et al. 2003; Zhou et al. 2003). As experience with this virus was accumulated, CPV was considered the benchmark sample for high-resolution study of single-particle cryo-EM. In 2008, Yu et al. reported the first high-resolution structure of BmCPV at 3.88 Å (Fig. 1B). More high-resolution structure of CPV then followed, including a 3.1-Å structure and a 3.9-Å structure of the more complete CPV (Cheng et al. 2011; Yu et al. 2011), and a cryo-EM structure of the transcribing CPV (Yang et al. 2012). However, the internal structures of genome and RdRps have been kept in the dark until Liu and Cheng’s work.

Aiming to build the structure of the genome and RdRps of CPV, the authors developed a so-called symmetry-mismatch reconstruction in their study. At the heart of the method, it finds out the accurate positional parameters for particles containing the genome and enzymes as a normal single-particle analysis dose (Frank 2009). The study took a two-step process to determine the parameters: first, making use of the icosahedral symmetry of the outer capsid shell, virions orientation searching was only limited to one asymmetric unit of the icosahedral space; second, to get the real azimuth of the genomes in each particle, here the first-step generated orientation was relaxed to full spherical space by allocating 60 possibilities, and then refined by iteratively aligning the interested inner part of each raw images with the references. The initial reference was obtained by masking the shell from the full virus map reconstructed using raw images with the random selection of one of the 60 equivalent orientations. During the second step, in order to eliminate the interference from the capsid and to reliably determine the azimuthal parameters, the authors used the capsid structure projection according to the particle orientation to mask the protein shell on the raw image by use of pixel subtraction, with the consideration of CTF correction and grayscale adjustment. The final structure was reconstructed from raw virus images other than the genome-extracted images using the determined positional parameters.

With their new strategy, Liu and Cheng solved the structures of both the NCPV and TCPV in the presented paper. The arrangements of genome and RdRps are alike in both virions, while the interactions between genomic RNA and RdRps differ in the two states. Their genome and RdRps adopts a pseudo-D3 symmetric organization, by which further reconstruction enhanced the resolutions to allow building a Cα model. Some extra densities are seen in the active sites in NCPV and TCPV and nucleotides can be assigned into them. From both maps, a fragment of dsRNA is observed to bind to each of the 12 RdRps bracelets, which shifts in NCPV compared to that in TCPV, which is interpreted as virion transcription at initiation stage. The channels for the exit of transcript and RNA template are partially blocked in NCPV RdRps, but completely open in TCPV RdRps. A loop in RdRps adopts a retracted conformation in NCPV and becomes extended in TCPV, thus it is interpreted as the switch loop. These details about the viral functional enzymes and genome are not seen in previous virus structure studies, which have been going on for nearly four decades (Harrison et al. 1978). The current work is therefore a landmark in structural virology.

On the basis of the internal structures of NCPV and TCPV plus previous knowledge on the *Reoviridae* viruses, Liu and Cheng proposed a dsRNA virus replication and transcription model, although structures of some potential states were still lacking in this study. According to their model, the replication initiates from the plus-strand RNA, which passes through the template entry channel of RdRps. The resulting dsRNA then exits at the template channel exit and binds to the bracelet domain of RdRps to prepare for transcription. Upon transcription, dsRNA disassociation from the binding site triggers the opening of the transcript and template exit channels, as well as the extension of the switch loop. The dsRNA is dissembled at the entrance of the template entry channel likely catalyzed by RdRps, and then the minus strand enters into the channel serving as template for synthesizing mRNA, while the plus strand stays outside the channel by binding its 5′ cap to the RdRps. When the finished transcript exits the transcript channel, the template associates with the original plus-strand RNA to form dsRNA for next round transcription. This four-channel coordinated model for dsRNA virus replication and transcription is a principle finding of Liu and Cheng’s structure analysis.

Regarding the methods, as the authors mentioned, similar idea had been used elsewhere including solving the bacteriophages local symmetry mismatched details (Tao et al. 1998; Morais et al. 2001; Jiang et al. 2006), and the kelp fly virus vertex (Briggs et al. 2005). A further usage of asymmetrical reconstruction named focused asymmetrical reconstruction was to reveal the tandem symmetry mismatches in T7 phage (Guo et al. 2013). However, neither did reach high resolution for these studies, nor were the genomes of these viruses solved. The authors also hinted that the methods used in their study are well suited to other macromolecules with mixed symmetry.

The current work’s data were collected on a CCD camera instead of a superior direct detector, which contributes a lot to image quality improvement and image processing (Liu and Zhang 2014). Thus, the resolutions of the NCPV and TCPV were not sufficient to allow the authors to build an accurate atomic model. Nevertheless, the work surely demonstrated that the genome is solvable by averaging a large amount of particles. The authors also admitted that the D3 symmetry in the dsRNA organization probably did not reflect the true arrangement given the fact that the RNA genome differs in size among the 10 segments in each virus.

Further application of direct detectors for data collection, combining with other image-processing algorithms, and improved specimen-preparation methods should bring other advances in the use of cryo-EM for structural virology. With the outstanding success of the current study, the gate is open for structural studies of internal contents of other viruses like phages and other infectious viruses (Dai et al. 2013; Guo et al. 2014; Serwer et al. 2014; Manokaran et al. 2015). Because viruses represent the simplest life-being and are the direct targets for anti-viral drugs, the findings may even unveil more fundamental mechanisms related to current biology and herald the dawn of single-particle cryo-EM as a method to facilitate rational anti-viral drug design.
